# The Effect of Cognitive Tasks on the Ocular Vestibular Evoked Myogenic Potentials in Healthy People

**DOI:** 10.22038/ijorl.2019.39322.2301

**Published:** 2020-09

**Authors:** Roya Sanayi, Vida Rahimi, Ghasem Mohamadkhani, Reza Hoseinabadi

**Affiliations:** 1 *Department of Audiology, School of Rehabilitation, Tehran University of Medical Sciences, Tehran, Iran.*

**Keywords:** Balance, Cognition, Ocular vestibular evoked myogenic potentials, Vestibular system

## Abstract

**Introduction:**

The majority of the daily life activities involve the concurrent performance of simultaneously challenging motor and cognitive activities, such as talking while walking, which requires the vestibular system for balance. Functional balance allows the brain to interpret and integrate the sensory information from our physical and social environment. This study aimed to investigate the effect of cognitive activities on the vestibular system function.

**Materials and Methods:**

This study investigated the otolith system as a sensory organ that is responsible for linear acceleration by recording ocular vestibular evoked myogenic potential (oVEMP) in 28 healthy participants (11 males and 17 females) with the age range of 18-26 years under a cognitive condition. The rest and intervention states were compared in terms of oVEMP n1-p1 amplitude, n1-p1 latencies, and gender.

**Results:**

The results showed that the oVEMP n1-p1 amplitude in both ears significantly decreased, and the asymmetry increased after cognitive tasks, compared to the rest state in females (P≤0.02). Moreover, there was no significant difference between the rest state and numeric subtraction task in terms of oVEMP n1-p1 latencies in males and females (P>0.05).

**Conclusion:**

These results suggest that an augmented cognitive load causes an alteration in the oVEMPs; therefore, it is suggested that the structures associated with the cognitive processing are connected with the vestibular system in the brain. These findings demonstrate the importance of non-vestibular factors in balance, especially in females.

## Introduction

The presentation of a high-intensity acoustic stimulus to an ear causes a sequence of reflexes, one of which is vestibular evoked myogenic potential (VEMP). This reflex could be recorded from cervical (cVEMP) or ocular (oVEMP) muscles.

In the cVEMP, a surface electrode is located on the sternocleidomastoid muscle; however, in the oVEMP, an electrode is located close to the inferior oblique muscle (1,2). The oVEMP is a test to evaluate the activity of the utricle and the inferior portion of the vestibular nerve. Moreover, this test activates the vestibular end organs. The signals in the vestibular nuclei connected with integration centers are located in the rostral midbrain tegmentum and thalamus leading to vestibular cortex areas (3). The vestibular cortex involves parieto-insular vestibular and medial superior temporal visual cortex (4). In addition to the connection between the vestibular system and other sensory and motor signals, this system interacts with different cognitive processes, such as spatial navigation (5), space perception (6), body representation (6,7), mental imagery (8-10), attention (11), memory (12), risk perception (13), and social cognition (14,15). 

Brandt et al. proposed a new classification that comprised the cognitive and other non-vestibular modalities (4). Several findings show that vestibular problems cause cognitive disorders that could be related to the reflexive deficits that are evidence of the interconnection between the limbic and neocortex structures with vestibular system (16). Therefore, cognitive processes can be one of the important factors affecting the results of the equilibrium tests. Some studies investigated the effect of cognitive processes on caloric and galvanic vestibular stimulation (C-GVS) (17,18). To our knowledge, CVS and GVS stimulate a different section of the vestibular organ, compared to VEMPs. The oVEMPs have been applied to diagnose and confirm the otolithic dysfunction and the inferior portion of the vestibular nerve (19). However, there is a dearth of research about the effect of cognitive load on oVEMPs (15). Therefore, this study aimed to investigate the effect of cognitive tasks on the function of the otolith organ and vestibulo-ocular reflex (VOR) in healthy young people without any complaint of vestibular or balance problems to determine the effect of top-down modalities of cognitive tasks on the otolith organ functions.

## Materials and Methods

In total, 28 healthy volunteers (11 males and 17 females) with the mean age of 22±3.13 years (age range: 18-26 years) participated in this study. Common otologic and neurologic tests (Videonystagmography [VNG]) and magnetic resonance imaging results were normal, and the presence of any problems in the central nervous system was ruled out by the physician report. Moreover, tympanometry and audiometry tests were performed thorough history taking and physical examination. 

The exclusion criteria were: 1) medical history of ear disease, 2) vertigo, 3) unconsciousness, 4) severe head trauma, 5) central nervous system disorders or proprioceptive dysfunction, and 6) vestibular diseases. The participants were also evaluated for anxiety and cognitive disorders. The study protocol was approved by the Ethics Committee of Tehran University of Medical Sciences, Tehran, Iran.

**Ocular Vestibular Evoked Myogenic Potential** **Procedure**

The volunteers underwent oVEMP recordings using the ICS Chartr EP 200 (Otometrics Inc., Denmark) with ER-3A insert earphones. The subjects were seated in an upright position. The ground electrode was located on the forehead. The non-inverting electrode was located 1 cm below the lower eyelid in the center position directly below the pupil, whereas the inverting electrode was located 1-2 cm under the non-inverting.The impedance of the electrode and the inter-electrode were <5k Ω and <2 kΩ, respectively. The insert earphone was placed in the ear contralateral to the eye for electrode placement. The participants were told to keep an upward gaze during the recording and gaze to a specific point on a wall. At first, the oVEMP was recorded in the resting state (stimulus, click; polarity, alternate; intensity, 125dbSPL; sweep,100; rate,5; gain, 40; filter, 5-500 Hz). Subsequently, a second oVEMP was recorded while the examiner asked the participant to perform certain cognitive tasks. Cognitive tasks in this study were similar to those used by Coelho et al. The subjects computed mental subtraction through a randomly determined number between 100 and 200 (20). Following that, the participant was asked to write the results of their mental calculations on the paper while still looking at the spot on the wall. In addition, the waves were recorded in the resting and cognitive task states separately and coded by a second examiner to avoid the examiner bias. The first negative-positive biphasic waveform included peaks n1 and p1. Repeated runs were performed to confirm the reproducibility of the peaks n1 and p1. The amplitude of n1-p1, asymmetry ratio, and latency of n1-p1were measured in this study (21).The data were analyzed in SPSS software (version 19.0), and a p-value less than 0.05 was considered statistically significant. The normality of data was checked using the Kolmogorov-Smirnov test. Moreover, two-way repeated measurement ANOVA was used to compare the rest and cognitive process modes by examining the effects of gender and ear (VEMP variables: gender and ear). Furthermore, pair sample t-test was employed to compare the variable results separated by ear and gender.

## Results

All 28 subjects showed oVEMPs in response to AC stimuli in two states. Table 1 tabulates the mean n1-p1 amplitude, n1 and p1 latencies, and asymmetry ratio. There were no significant differences between oVEMP in a rest state and oVEMP during the mental calculations in terms of the n1-p1 amplitudes, asymmetry ratio, and n1-p1 latencies (P≥0.05). 

However, there were significant differences between oVEMP in rest state and oVEMP during the mental calculations in terms of n1-p1 amplitudes and gender (P<0.02). Moreover, the mean values of n1-p1 amplitude in the right (5.70±2.9) and left ears (6.43±3.8) were significantly reduced (5.01±2.54 and 5.39±4, respectively) during the mental calculations. Furthermore, there was a difference between the results of the two states regarding the asymmetry ratio which showed an increase in this regard (P<0.02); however, the mean n1-p1 latencies were not significant (P≥0.05). 

**Table1:** Comparison of the rest state and during a cognitive process mode regarding mean±SD of amplitude, latency, and asymmetry waves and ANOVA results

Variable	Rest	During a cognitive process	F	P-value
**Amplitude**	6.07±3.42	5.20±3.36	0.85	0.35
**Latency p1**	15.02±0.98	15.14±1.07	3.34	0.073
**Latency n1**	10.26±0.89	10.55±1.26	0.13	0.71
Asymmetry(*gender)	18.17±13.89	27.09±15.24	3.41	0.075

**Fig 1:**
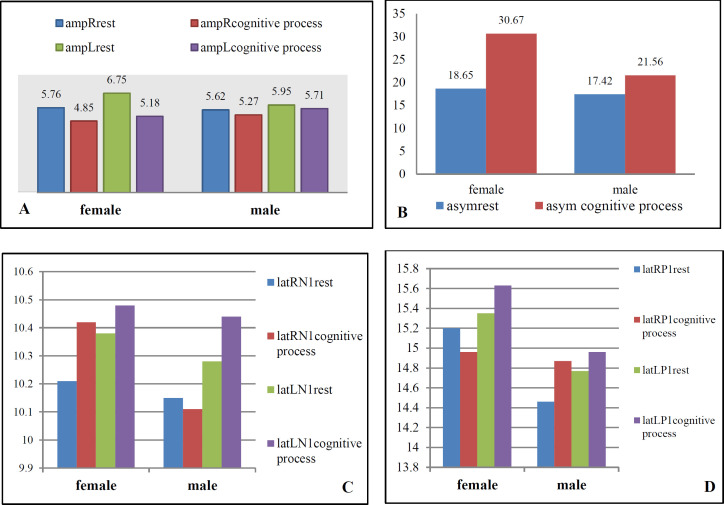
Mean n1-p1 amplitude in both ears in rest and during a cognitive process mode regarding gender

Table 2 summarizes the p-values and the mean±SD of amplitude, latency, and asymmetry waves in both ears. Statistical analysis showed different results in terms of amplitude and asymmetry based on gender. In females, the mean n1-p1 amplitude in both ears significantly reduced by adding a cognitive process (P≤0.02). Moreover, the asymmetry ratio was increased in the intervention state (P=0.000) (Fig. 1). Surprisingly, no significant differences were observed between the two states in terms of amplitude and asymmetry in males (P>0.2). Similarly, there was no significant difference between the rest state and during mental calculations regarding the mean difference between the n1-p1 latencies in both groups (P>0.08).

**Table2:** Mean of amplitude, latency, and asymmetry waves in both ears at rest and during a cognitive process

Variable		Rest	During a cognitive process	F	P-value
**Amplitude**	Right ear	5.70±2.99	5.01±2.54	6.87	0.014
	Left ear	6.43±3.82	5.39±4.06	9.77	0.004
**Latency p1**	right ear	14.91±.87	14.92±.88	0.5	0.94
	left ear	15.12±1.08	15.37±1.21	2.8	0.052
**Latency n1**	right ear	10.19±0.73	10.30±1.01	.004	0.48
	left ear	10.34±1.34	10.80±1.44	4.23	0.14
**asymmetry**		18.17±13.89	27.09±15.24	17.01	0.000

**A) **ampRrest=n1-p1 amplitude of oVEMP in the right ear in a rest state, ampR cognitive process=n1-p1 amplitude of oVEMP in the right ear during a cognitive process, ampLrest= n1-p1 amplitude of oVEMP in the left ear in a rest state, ampL cognitive process= n1-p1 amplitude of oVEMP in the left ear during a cognitive process.

**B) **asym rest=asymmetry ratio of oVEMP in a rest state. asym cognitive process= asymmetry ratio of oVEMP during a cognitive process**.**

**C)** LatRN1rest=N1 latency in the right ear in a rest state. LatRN1cognitive process=N1 latency in the right ear during a cognitive process. latLN1rest=N1 latency in the left ear in the rest state. LatLN1cognitive process=N1 latency in the left ear during a cognitive process**.**

**D)** LatRP1rest=P1 latency in right ear in rest state.latRP1cognitive process=P1 latency in right ear during a cognitive process. latLP1rest=P1 latency in left ear in rest state.latLP1cognitive process=P1 latency in left ear during a cognitive processing

## Discussion

This study aimed to investigate the effect of cognitive processing on the oVEMP. The oVEMP, analogous to VOR, can be recorded from the extraocular muscles by a surface electrode under the contralateral infraorbital margin. Formerly, VEMPs were used as diagnostic tools in peripheral disorders; however, recently, they are increasingly used in central and cognitive levels in the field of balance. According to the data suggested by Talkowski et al., VOR and ocular motor systems are not completely automatic systems even though they are associated with cognitive resources. This relation occurs with cognition as a result of the sensory integration in dealing with the inputs from multiple sensory routes and a continuous cognitive resource is needed to compare the unilateral vestibular loss (22).

In this study, numeric subtraction was utilized as a cognitive task according to a study by Coelho s et al. (20). Although no difference was observed between the results in the rest and the cognitive modes, the findings were different when gender was taken into account. The decreased amplitude of n1-p1 in both ears in the cognitive state was significant in females. Moreover, oVEMP asymmetry increased during the cognitive process, and this was also prominent in females. However, there was no significant difference between the two conditions in males and females regarding waves p1-n1 latencies. It seems that the vestibular otolith-ocular function reduces along with the cognitive task, and it will be unfavorable because this diminution of vestibular otolith-ocular function is related to increased mediolateral sways which leads to an increase in the risk of falls (23).

Recently, a systematic review performed by Muir-Hunter and Wittwer under dual-task conditions showed a prominent association between the diminution of gait and the probability of falls (24). Furthermore, Hall, Echt, Wolf, and Rogers revealed that the effect of the second cognitive task was more than the motor activity on the alterations in the gait kinematics (25).

In addition to the behavioral studies, brain imaging studies illustrate the activation of the regions that are related to higher cognitive control during the actual, imagined, and simulated gait. In the same line, lesion behavior studies narrate the considerable impact of higher cognitive control systems on gait control. There is evidence that confirming posture control and cognitive tasks have the same command areas. 

These results were obtained by modulating the impacts of concurrent postural and cognitive functions (26). Regarding how the cognitive load affects the balance system, previous studies demonstrated that the cognitive load causes neuromuscular changes, such as increased intra-cortical inhibition (27).

The reason why increased cognitive processes disturb the motor task operation is that whenever the processing demands are increased through a secondary cognitive task (28,29), these systemic constraints increase and cause the poorer performance of one or both tasks (28). 

McGeehan et al. stated that an increase in the cognitive load caused the enhancement of the vestibular control of balance standing which was inconsistent with the results of this study. The increased neural plan may be executed to provide separate cortical processing sources within the balance system and recompense for the acute neuromuscular rectification related to the augmented cognitive request (30). 

As illustrated in this study, cognitive load causes a decrease in the amplitude and an increase in the asymmetry, which was significant in females, not males. McGeehan et al. demonstrated that in a dual-task paradigm, some factors consisted of the difficulty of the motor and cognitive tasks or convenience of both tasks for participants (30). Since no studies investigated the cognitive effects on the results of the oVEMP, the observed difference can be due to the difference in the type of assessment in the study performed by McGeehan versus this study (anterior-posterior ground-body forces vs oVEMP). 

Therefore, they evaluated the compensatory mechanisms for balancing equilibrium (30). Furthermore, studies showed that an increase in cognitive demand could have an effect on the VOR responses (30), and changes in the VOR responses could affect the responses of oVEMP (31). Regarding the observed differences between males and females, these studies suggest that there are gender differences in cognitive function, and sex hormones seem to influence cognitive performance. For instance, males outperform females in mathematical problem solving (32). 

Therefore, it can have different effects on the results of vestibular tests. In addition, the limited number of male samples can be one of the reasons for not being effective. Nevertheless, studies about the effect of gender through cognition on the vestibular function are few, and more surveys are required in this regard. Additionally, the reason for an increase in asymmetry in female samples is not clear; therefore, there is a need for further studies in this area.

## Conclusion

The findings of this study depict that in the cognitive state, the oVEMP n1-p1 amplitude in both ears decreased along with an increase in the asymmetry. Increased cognitive load causes the oVEMPs alteration, and therefore, demonstrates evidence that the structures associated with the cognitive processing are connected with the vestibular system in the brain. These results were significant in females and support the significance of non-vestibular factors in balance, especially in females. However, further studies are required in this regard. 
